# Comparative Genomics of *Limosilactobacillus reuteri* YLR001 Reveals Genetic Diversity and Probiotic Properties

**DOI:** 10.3390/microorganisms12081636

**Published:** 2024-08-10

**Authors:** Lihong Zhang, Md. F. Kulyar, Tian Niu, Shuai Yang, Wenjing Chen

**Affiliations:** 1 College of Veterinary Medicine, Gansu Agricultural University, Lanzhou 730070, China; nt18393170049@163.com (T.N.); yangshuai11200@163.com (S.Y.); 13993117762@163.com (W.C.); 2 College of Veterinary Medicine, Huazhong Agricultural University, Wuhan 430070, China; fakharealam786@hotmail.com

**Keywords:** *Limosilactobacillus reuteri*, complete genome sequencing, probiotic properties, yak

## Abstract

To gain deeper insights into the genomic characteristics of *Limosilactobacillus reuteri* (*L. reuteri*) YLR001 and uncover its probiotic properties, in the current study, a comprehensive analysis of its whole genome was conducted, explicitly exploring the genetic variations associated with different host organisms. The genome of YLR001 consisted of a circular 2,242,943 bp chromosome with a GC content of 38.84%, along with three circular plasmids (24,864, 38, 926, and 132,625 bp). Among the 2183 protein-coding sequences (CDSs), the specific genes associated with genetic adaptation and stress resistance were identified. We predicted the function of COG protein genes and analyzed the KEGG pathways. Comparative genome analysis revealed that the pan-genome contained 5207 gene families, including 475 core gene families and 941 strain-specific genes. Phylogenetic analysis revealed distinct host specificity among 20 strains of *L. reuteri*, highlighting substantial genetic diversity across different hosts. This study enhanced our comprehension of the genetic diversity of *L. reuteri* YLR001, demonstrated its potential probiotic characteristics, and established more solid groundwork for future applications.

## 1. Introduction

Many old species of *Lactobacillus* have been reclassified into new genera according to recent taxonomic revisions. One of these is the species formerly known as *Lactobacillus reuteri* but now referred to as *Limosilactobacillus reuteri* (*L. reuteri*). This reflects a greater understanding of the phylogenetic relationships within the former *Lactobacillus* genus [1]. *L. reuteri*, a Gram-positive and nonmotile bacterial species, is a heterofermentative LAB microorganism that commonly resides in the gastrointestinal (GI) tract of humans and other vertebrate animals such as mice, rats, pigs, chickens, and cows [2]. In recent years, strains of *L. reuteri* have demonstrated diverse probiotic effects, including their ability to colonize the intestine [3], modulate the host immune system [4], produce and secrete a wide range of antimicrobial compounds [5], prevent diarrhea [6], and mitigate colitis [7]. Moreover, *L. reuteri* exhibits remarkable resistance to acidity levels, bile salts, and osmotic stress. Therefore, understanding the potential probiotic properties of *L. reuteri* sheds light on their evolutionary adaptations and possible implications.

As a valuable model microorganism for understanding the evolution of animals’ gut symbionts, extensive research and investigation have been conducted to explore the growth characteristics, metabolism, and evolutionary patterns of *L. reuteri* [8]. Son et al. conducted a comparative genomics analysis to characterize a canine-hosted strain of *L. reuteri*, revealing striking similarities with human-associated strains [9]. In another study by Yu et al., the genetic diversity of *L. reuteri* strains obtained from herbivorous hosts such as goats, sheep, cows, and horses was assessed. The findings unequivocally demonstrated that these host-specific *L. reuteri* strains exhibit remarkable genetic diversity linked to their respective ecological niches [10]. In addition, ecological investigations have provided valuable insights into the intricate dynamics of *L. reuteri* within its host organism and other microorganisms residing in the gastrointestinal tract [11]. It has been revealed that *L. reuteri* plays a crucial role in maintaining gut health by facilitating digestion, modulating immune responses, and preventing colonization by harmful pathogens. Moreover, the genomic approach and animal model experiments have been employed to analyze the evolution and ecology of *L. reuteri* in the gastrointestinal tract of animals, thereby providing further validation for adapting host-specific strains [12]. However, the evolutionary strategies employed by *L. reuteri* strains isolated from Yak’s GI tracts remain unknown, as do the ecological relationships between yak intestinal isolates and other intestinal isolates.

A yak (Bos mutus)-derived strain of *Limosilactobacillus reuteri* YLR001, which exhibited numerous probiotic properties, was identified based on its morphology, physiological, and chemotaxonomic properties. Our previous studies showed positive effects on growth performance, intestinal microbiota composition, and antioxidant status in weaned yaks when supplementing their diet with the probiotic additive containing *L. reuteri* YLR001 [13]. To gain further insights into the genetic adaptation of *L. reuteri* YLR001 to the host gut and understand its genome diversity and phylogenetic evolution status, a comparative genomic approach was employed to assess its genetic diversity and probe its potential probiotic-related genetic traits. These findings provide valuable knowledge regarding strains’ evolutionary processes and adaptive mechanisms in specific environments.

## 2. Materials and Methods

### 2.1. Bacterial Growth and Genomic DNA Extraction

*L. reuteri* YLR001 was isolated from the gastrointestinal mucosa of a healthy yak and identified through colony morphology, physiological and biochemical experiments, and 16S rRNA sequence analysis. *L. reuteri* YLR001 was cultivated in de Man Rogosa Sharpe broth (MRS broth, Hopebio, Qingdao, China) or MRS agar under aerobic conditions at 37 °C. Genomic DNA was extracted and purified using the Wizard^®^ Genomic DNA Purification Kit (Promega, Madison, WI, USA), following the manufacturer’s instructions [14]. The harvested DNA was analyzed by agarose gel electrophoresis and quantified using a TBS-380 fluorometer (Turner BioSystems Inc., Sunnyvale, CA, USA).

### 2.2. Whole-Genome Sequence Analysis of L. reuteri YLR001

Whole-genome sequencing was conducted by Beijing Novogene Bioinformatics Technology Co., Ltd. (Beijing, China) using a combination of PacBio RS II Single Molecule Real Time (SMRT) and Illumina sequencing platforms. The complexity of the genome was evaluated using Illumina data. For Illumina sequencing, a minimum of 1 μg of genomic DNA was utilized for each strain in the construction of sequencing libraries. The DNA samples were fragmented into 400–500 bp fragments using a Covaris M220 Focused Acoustic Shearer according to the manufacturer’s protocol. Subsequently, Illumina sequencing libraries were prepared from the fragmented DNA using the NEXTflex™ Rapid DNA-Seq Kit. For Pacific Biosciences sequencing, 15 μg of DNA was processed in a Covaris g-TUBE (Covaris, Woburn, MA, USA) at 6000 RPM for 60 s using an Eppendorf 5424 centrifuge (Eppendorf, Hauppauge, NY, USA). DNA fragments were then purified, end-repaired, and ligated with SMRTbell sequencing adapters following the manufacturer’s recommendations (Pacific Biosciences, Menlo Park, CA, USA). The genome library was constructed and sequenced using standard methods on one SMRT cell. PacBio and Illumina platform-generated data were used for bioinformatics analysis. Herein, we assembled the complete genome sequence to a coverage of 100× using SOAPdenovo version 2.04 for data generated by the Illumina platform [15,16,17].

### 2.3. Genome Annotation of L. reuteri YLR001 

The coding sequences (CDS) were predicted using Glimmer v. 3.02b software, while tRNA and rRNA were identified using tRNA-scan-SE and Barrnap v. 0.9 software, respectively. To assess the probiotic potential of *L. reuteri* YLR001, CDSs were annotated from NR, Swiss-Prot, Pfam, GO, COG, and KEGG databases via sequence alignment tools such as BLAST, Diamond, and HMMER. To assess *L. reuteri* YLR001 for its antibiotic resistance potential, its genome was screened for the presence of antibiotic resistance genes in the Comprehensive Antibiotic Resistance Database (CARD). The analysis was performed independent of our general annotation process, in which functional gene annotation was taken against NR, SwissProt, Pfam, GO, COG, and KEGG databases. Additionally, virulence factors were predicted by utilizing the Virulence Factors Database (VFDB) [18,19].

### 2.4. Comparative Genomics Analysis of the Lactobacillus Family

To evaluate the genetic relatedness between YLR001 and the *L. reuteri* family, 19 genomes of *L. reutrei* strains were obtained from the NCBI database (Supplementary Table S1). The phylogenetic tree was constructed using Mega X software (MEGA 11 version 11.0.13) employing the Neighbor-Joining method. All predicted protein sequences were merged and subjected to BLASTP algorithm-based comparisons for comparative analysis of functional gene contents. Average nucleotide identification (ANI) was determined using the JSpecies web server. An orthologous gene set was established utilizing OrthoMCL package v2.0 to identify core-genome and pan-genome sizes [20].

Homologous protein pairs were clustered into orthologous families using the MCL tool with an inflation value of 1.5, followed by alignment of predicted amino acid sequences for each single copy orthologous gene family using MAFFT v7. The resulting individual alignments were concatenated to generate a string of amino acid sequence alignment data, which was then submitted to RAxML for phylogenetic tree construction via the maximum likelihood algorithm. All analyses were performed using the free online platform of Majorbio Cloud Platform (www.majorbio.com: accessed on 13 December 2023).

### 2.5. Data Accession Number

The raw and assembled sequence data for the *L. reuteri* YLR001 genome have been deposited at SRA databases under the accession number PRJNA682320 (SAMN16987598) and GenBank under the accession numbers CP065540 (chromosome), CP065541 (plasmid1), CP065542 (plasmid2), and CP065543 (plasmid3).

## 3. Results and Discussion

### 3.1. Genome Features of L. reuteri YLR001

The reclassification of *Lactobacillus reuteri* as *Limosilactobacillus reuteri* underlines the increasing understanding of the genetic diversity within the former *Lactobacillus* genus. In view of such a taxonomic update, this study points to the peculiarity of *L. reuteri* among the former *Lactobacillus* species. The genomic analysis of *L. reuteri* YLR001 performed in this study has contributed to understanding this newly defined genus and, therefore, underlined the specific adaptation and probiotic properties that characterize *Limosilactobacillus* species [1]. The main genomic features that were discovered about *L. reuteri* YLR001 are summarized in Table 1, providing a comprehensive overview of its genetic composition. The complete genome of *L. reuteri* YLR001 consists of a single circular chromosome spanning 2,242,943 bp with a GC content of 38.84%. In addition to the chromosome, this strain also harbors three plasmids: plasmid 1 (132,625 bp with a GC content of 36.39%), plasmid 2 (38,926 bp with a GC content of 39.26%), and plasmid 3 (24,864 bp with a GC content of 33.51%).

Within the chromosome, our analysis predicted the presence of various functional elements, including protein-coding genes (CDSs), tRNA genes for translation processes, rRNA genes involved in ribosome assembly and function, and the sRNA gene responsible for regulatory functions. Specifically, 2183 protein-coding genes within the *L. reuteri* YLR001 genome were identified. These genes play crucial roles in encoding proteins that carry out diverse biological functions essential for the survival and growth of this bacterium. Furthermore, the analysis revealed the presence of 69 tRNA genes, which facilitate accurate translation by matching specific amino acids to their corresponding codons during protein synthesis. The 18 rRNA gene numbers within the genome structure were further identified. These rRNAs contribute to ribosome formation and ensure efficient protein production. Additionally, one sRNA gene was identified in the chromosome region, which is known to regulate gene expression through post-transcriptional mechanisms.

**Figure 1:**
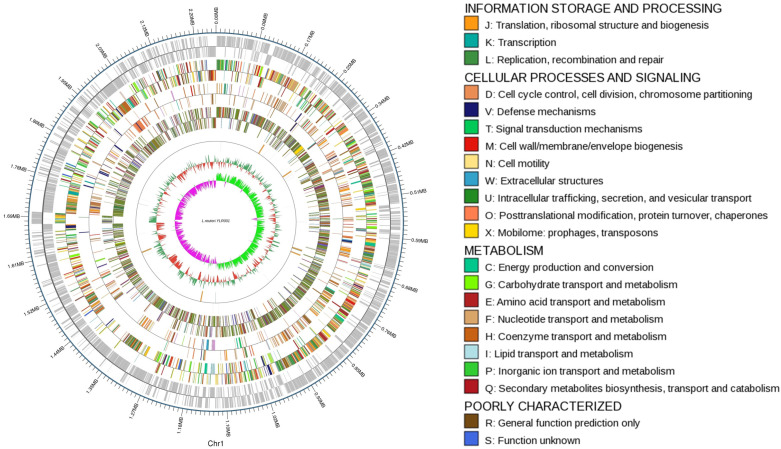
Circular genome map of *L. reuteri* YLR001.

A circular genome map has been generated to visually represent these findings and provide an overall view of how these genomic features are distributed across the *L. reuteri* YLR001 circular genome structure (Figure 1).

### 3.2. Functional Classification

**Figure 2:**
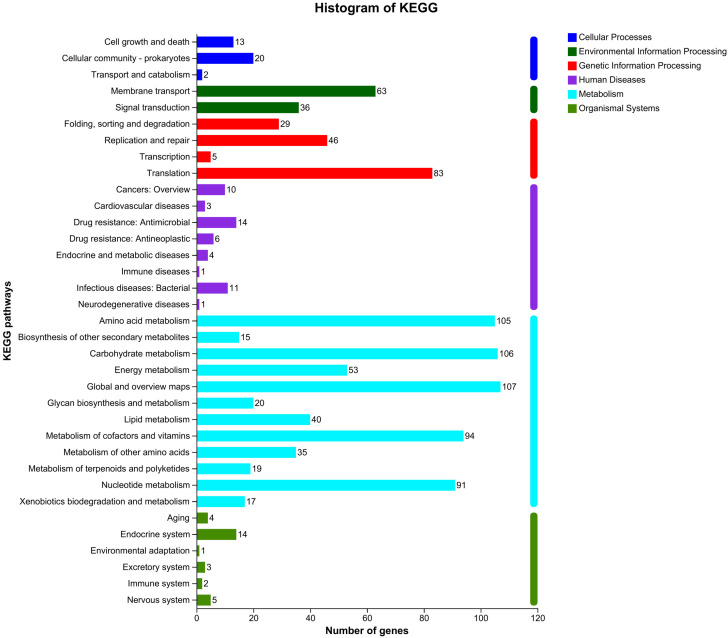
KEGG classification statistics of the *L. reuteri* YLR001 genome annotation.

A total of 1134 CDSs were classified into 35 KEGG functional categories, mainly functioning in global and overview maps, carbohydrate metabolism, amino acid metabolism, metabolism of cofactors, vitamins, and nucleotide metabolism (Figure 2). Within the KEGG functional categories, it was observed that a considerable proportion of genes played crucial roles in global and overview maps (107 genes). Moreover, KEGG analysis revealed mappings to many pathways to human diseases. Although these categories may perhaps seem unrelated to bacterial functions, they reflect both the comprehensiveness of the KEGG database and the possible functional analogy between the bacterial genes and eukaryotic pathways. The mappings in no way reflect a direct association with human diseases but suggest possible functionalities or host–microbe interaction pathways. Also, the involved genes provide an overall understanding of complex biological pathways and networks within the organism.

Carbohydrate metabolism is a critical area where numerous genes (106 genes) were identified. These genes break down carbohydrates into simpler molecules for energy production or storage. Similarly, amino acid metabolism plays a vital role in synthesizing essential building blocks for proteins and other important biomolecules (105 genes). Additionally, our analysis revealed a significant presence of genes (94 genes) associated with the metabolism of cofactors and vitamins. These genes facilitate various enzymatic reactions by acting as coenzymes or cofactors. Furthermore, nucleotide metabolism is fundamental for DNA replication, RNA synthesis, and cell division. Identifying many genes (91 genes) associated with each metabolic pathway emphasizes their critical role in sustaining life processes.

**Figure 3:**
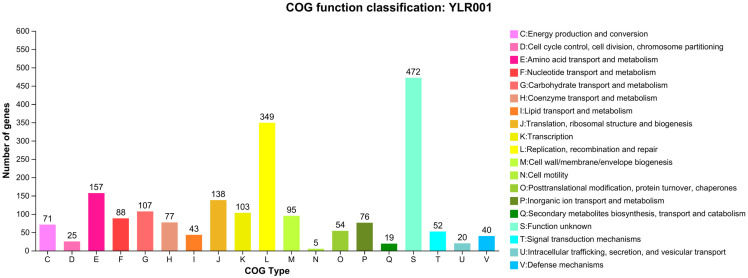
COG function classification of the *L. reuteri* YLR001 genome annotation.

The 1964 CDSs were found to be specifically assigned to these groups across 19 different categories (Figure 3). Among these categories, replication, recombination, and repair stood out, with many genes (349 genes) classified within them. This emphasizes the significance of maintaining genetic stability through accurate DNA replication and efficient repair mechanisms. Amino acid transport and metabolism also emerged as prominent COG categories, containing numerous genes (157 genes) involved in amino acid uptake from external sources and subsequent utilization within cellular processes like protein synthesis. Additionally, translation-related functions represented by ribosomal structure biogenesis showed substantial gene (138 genes) representation within COG clusters, indicating their critical role in protein synthesis machinery. Moreover, carbohydrate transport and metabolism appeared as another essential category (107 genes), highlighting their involvement in nutrient acquisition from external sources and subsequent metabolic processing inside cells. Lastly, transcription-related functions exhibited notable gene representation (103 genes), suggesting their pivotal role in regulating gene expression patterns necessary for proper cellular functioning.

#### Nitrogen Metabolism Genes and Their Functions in the Probiotic Properties of *L. reuteri* YLR001

Several genes identified from genome analysis also played definite roles in the probiotic properties of *Limosilactobacillus reuteri* YLR001. Of these, most genes fell into the KEGG functional categories “Amino acid metabolism”, “Metabolism of other amino acids”, and into the COG category “Amino acid transport and metabolism”. A significant number of 105 genes were linked to amino acid metabolism in the KEGG analysis, thus indicating that nitrogen-related processes are highly involved in *L. reuteri* YLR001. A number of involved pathways resulting in the production, degradation, and conversion of amino acids play a high role in proteins’ formation and other cellular functions [21].

The COG analysis provides further support that nitrogen metabolism genes are present. This includes genes coding for enzymes like glutamate dehydrogenase (GDH), glutamine synthetase (GS), and multiple amino acid permeases [10]. GDH is another enzyme that participates in the reversible oxidative deamination of glutamate to form α-ketoglutarate and ammonia, further playing a central role in nitrogen assimilation. The presence of GDH in *L. reuteri* YLR001 presumably confers its ability to utilize ammonia for glutamate synthesis, which is an amino acid crucial for bacterial growth and metabolism. GS is an ATP-dependent enzyme that converts glutamate to glutamine and ammonia. This is an enzyme that catalyzes the reaction responsible for nitrogen assimilation because it provides a source of nitrogen needed to make other amino acids and nucleotides. Having this enzyme in the genome of *L. reuteri* YLR001 ensured its potential ability to assimilate nitrogen efficiently and to support cellular functions under nitrogen-starved conditions.

The high count of genes in category “Amino acid transport and metabolism” suggests the existence of several amino acid permeases, providing *L. reuteri* YLR001 with the potential to scavenge amino acids from its environment. The identified capability is highly advantageous within the fiercely competitive environment of the gastrointestinal tract [22]. The role of nitrogen metabolism towards the probiotic activity in *L. reuteri* YLR001 is therefore invaluable. Effective nitrogen assimilation and amino acid biosynthesis are examples of the way bioactive peptides are derived, which already possess a variety of health benefits. These bioactive peptides can display antimicrobial, immunomodulatory, and antihypertensive characteristics, adding value to *L. reuteri* YLR001 [2,23]. Synthesis of essential amino acids and other nitrogenous compounds within the human small intestine fosters gastrointestinal growth and colonization of *L. reuteri*, further promoting good gut health and preventing colonization by such bacteria.

Hence, the nitrogen metabolism pathways identified by KEGG and COG functional categories in *L. reuteri* YLR001 are therefore relevant to its probiotic properties in sustaining growth, survival, and bioactive peptide production. These results may therefore be critical for a better understanding of the functional capacities of LAB and also their interest as probiotics.

### 3.3. Phylogenetic Relationships among L. reuteri Strains

**Figure 4:**
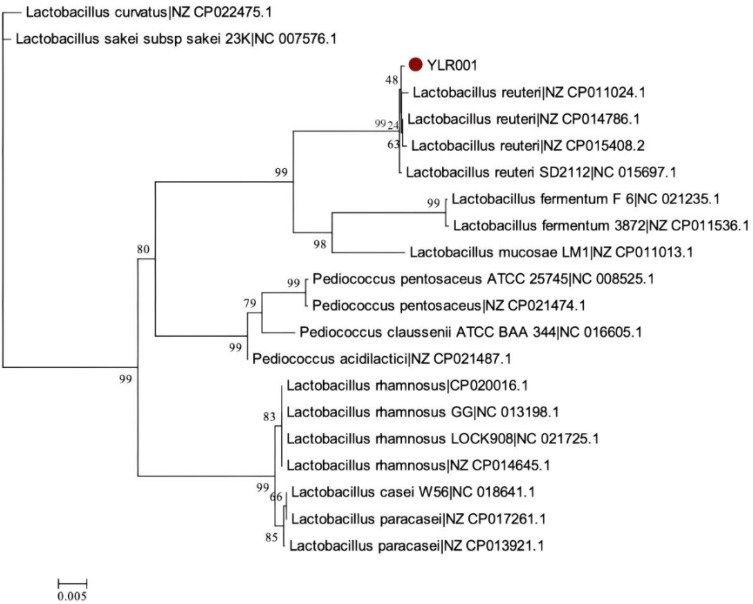
Phylogenetic tree of *L. reuteri* YLR001. The tree was constructed using MEGA 6.0 by the Neighbor-Joining method based on 16S rRNA gene sequences with 1000 replications in a bootstrap test.

The phylogenetic tree was constructed in MEGA 6.0 software using the Neighbor-Joining method, based on 16S rRNA gene sequences. A bootstrap test with 1000 replications was generated to ensure that the inferred phylogeny is reliable to understand the patterns of genomic evolution for unraveling how different *L. reuteri* strains have adapted to diverse ecological niches within the gut microbiome. Moreover, the analysis was undertaken to depict the broader phylogenetic relationships within the *Lactobacillus* group. The ability of certain strains to establish themselves as dominant members of this microbial community may be attributed to specific genomic features or functional traits encoded by their genomes. The phylogenetic tree shows that *L. reuteri* YLR001 revealed the genetic evolution between different *L. reuteri* strains (Figure 4). This analysis provides valuable insights into the evolutionary relationships and genetic diversity within the *L. reuteri* species. The phylogenetic tree demonstrates that *L. reuteri* YLR001 forms a distinct branch along with strains IRT, indicating their close genetic relation. This suggests a common ancestry or recent divergence among these strains, possibly due to shared ecological niches or similar selection.

**Figure 5:**
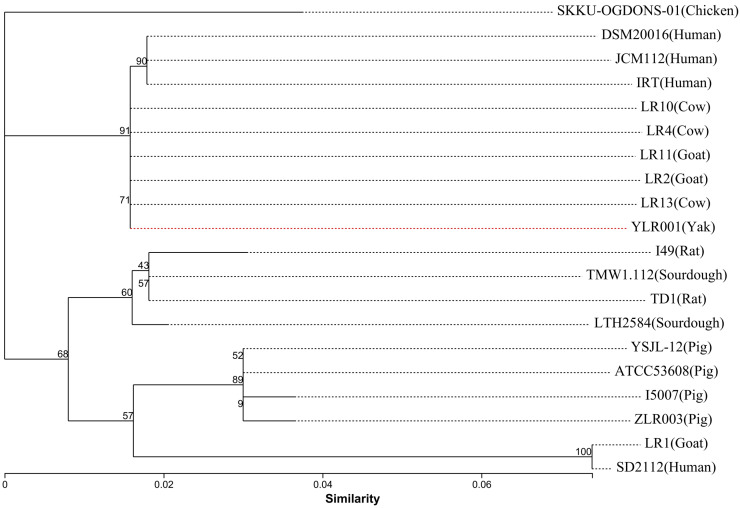
Phylogenetic tree of twenty *L. reuteri* strains. The phylogenetic tree was constructed based on the core gene families. The bootstrap support value before each node represents the confidence degree of each branch. The red dotted line in the figure highlights the bacterial strain under observation.

To further explore the phylogenetic relationship among *L. reuteri* strains, a comprehensive analysis was conducted based on the core gene families. In total, 475 core orthologous gene families were identified, providing a robust dataset for inferring evolutionary relationships. It was observed that SD2112 (human) and LR1 (goat) were assigned to a monophyletic group, and other strains were assigned to another monophyletic group (Figure 5). Interestingly, *L. reuteri* YLR001 exhibited a close relationship with strain LR13 based on this analysis. However, it is worth noting that YLR001 occupies its distinct branch within the phylogenetic tree. This observation suggests that strain YLR001 has undergone unique genomic changes or adaptations to thrive better in its specific gut environment. These findings shed light on both microevolutionary processes occurring within individual lineages of *L. reuteri* and macroevolutionary trends shaping diversification across multiple lineages within this species group. Further research is warranted to investigate the functional implications of these observed genetic variations and their potential impact on host–microbe interactions in various gut environments.

### 3.4. Core- and Pan-Genomes of L. reuteri Strains

**Figure 6:**
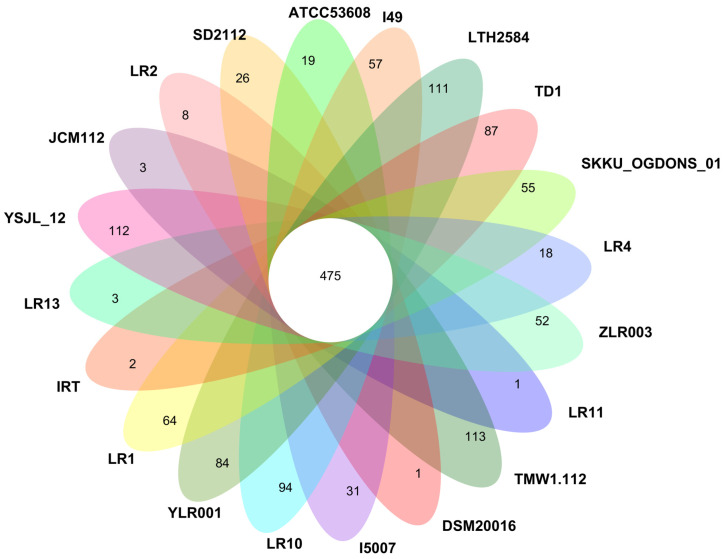
Numbers of orthologous gene families and unique genes among 20 *L. reuteri* strains. The different colors in the figure show different bacterial strains that were observed.

OrthoMCL analysis revealed the genomes of *L. reuteri* members. The pan-genome size was determined to be 5207 orthologous gene families (Figure 6), indicating a diverse genetic pool among these strains. This suggests that these organisms possess an open pan-genome structure, allowing new genes and genetic variations to be incorporated. Furthermore, it was observed that 475 (9.12%) orthologous gene families were present in all 20 genomes analyzed, representing the core genome of *L. reuteri* members. These core genes are likely essential for basic cellular functions and shared characteristics among different strains. In addition to the core genome, many strain-specific genes were identified within each strain. 941 (18.07%) strain-specific genes were found across all genomes studied, ranging from just one unique gene in strain DSM20016 to as many as 113 unique genes in strain TMW1.112.

Notably, it was discovered that the YLR001 strain possessed 84 unique genes not found in any other *L. reuteri* member analyzed in this study (Table S2). The presence of such a large gene pool and numerous strain-specific genes highlights the adaptability and versatility of *L. reuteri* members. This genetic diversity allows them to thrive and survive in various environments or interact with different hosts effectively.

These findings shed light on the genomic complexity and plasticity within *L. reuteri* species while emphasizing their ability to adapt and evolve according to their surroundings or host interactions.

### 3.5. Carbohydrate Metabolism and Transporter

Carbohydrate metabolism is a crucial process in *lactobacillus*, providing the primary source of metabolic energy and contributing to their ecological fitness [24]. This metabolic pathway involves various transporters, including the phosphotransferase system (PTS) and ATP-binding cassette (ABC) transporter systems. The PTS system, widely distributed in bacterial cells, is significant in transporting multiple carbohydrates into the bacterial cell [25]. It has several components, including enzyme I (EI), histidine protein (HPr), and enzyme II complexes specific to different sugars. These enzymes work together to phosphorylate incoming sugars during transport, allowing them to be efficiently metabolized within the cell.

Within these carbohydrate transporters, we have identified four genes encoding key components of the PTS system in *lactobacillus* (Table S3). The first gene is *celB* (gene0268), which encodes an enzyme II complex responsible for transporting cellobiose into the bacterial cell. Cellobiose is a disaccharide derived from cellulose degradation and is an essential *lactobacilli* carbon source. The second gene we have identified is *gatC* (gene 1967). This gene encodes another enzyme II complex that transports galactitol into *lactobacillus* cells. Galactitol is commonly found in dairy products and plant tissues, making it an abundant substrate for *lactobacilli* residing in such environments. Analysis revealed the presence of ptsI both on the chromosome (gene01576) and on plasmid B (gene 0033). PtsI is a gene encoding for the EI component of the PTS system, introducing the phosphate in the sugar during its membrane transport. Its duplication on both genetic elements probably mirrors its importance in carbohydrate metabolism in different environments. This would mean that *Limosilactobacillus* could metabolize a wide variety of carbohydrates, hence balancing the ecosystem.

In the genome of YLR001, 50 genes associated with the genomic ABC transporter system have been identified, such as abcA, cydD, and cydC (gene 0023, gene0575, and gene0576), respectively; all are key components in carrying molecules across membranes for cell homeostasis. The genes encoding cobalt/nickel transport proteins cbiO, cbiQ, cbiM, and cbiN, and the cystine transport system fliY, were identified, suggesting mechanisms for YLR001 to acquire essential trace elements and amino acids. Besides, nine genes involved in the D-methionine transport system were identified, such as metI, metN, metQ, and rbsD, which may help to take up D-methionine for protein synthesis. In the case of the energy-coupling factor transport system, ecfT, ecfA2, and ecfA1 were found, which exploit the energy derived from ATP hydrolysis for active transport. The iron complex transport system, which is key to the assimilation of iron ions, and the spermidine/putrescine transport system with the potA, potB, potC, and potD genes testify to the potential of YLR001 to acquire key molecules for its diverse cellular activities. In addition, the genome of *L. reuteri* YLR001 also contained eight major facilitator superfamily transporters. These transporters play a crucial role in facilitating the movement of various molecules across cell membranes [26], suggesting that *Limosilactobacillus* has developed an enhanced ability to uptake and utilize carbon sources.

The current analysis brings insight into the genomic features and probiotic properties of *L. reuteri* YLR001. The complete genome consisted of a circular chromosome of 2,242,943 bp and three plasmids, whereas a total of 2183 protein-coding sequences were predicted. Genes related to carbon metabolism, including glycolysis and the pentose phosphate pathway, which support efficient energy conversion and adaptation in the gastrointestinal tract, were identified. In addition, the Sec-SRP secretion system was identified, responsible for protein translocation and components that include, among others, the SRP protein ffh, gene 1328, and the SRP-docking protein FtsY, gene 1330. These elements are involved in correct protein targeting and translocation, a process very critical to cellular activity. Functional classification showed significant activities of carbohydrate metabolism and amino acid metabolism, under which 1134 CDS were classified into 35 KEGG functional categories. Phylogenetic analysis indicated genetic variation within *Limosilactobacillus*. It provided very strong support for the *L. reuteri* strains, including YLR001, where it formed a distinct branch, and thus their common ancestry and ecological adaptations were proven. These findings further improved the current knowledge of genetic diversity in *L. reuteri* and its potential use as a probiotic.

Understanding the genetic basis underlying the adaptation characteristics of YLR001 to the GI tract is essential not only from a fundamental research perspective but also for potential applications in probiotic development or gut health improvement strategies. Further studies investigating how these transporter systems are regulated under different conditions will provide valuable insights into their functional roles within *Limosilactobacillus* species and their interactions with host organisms. These findings highlight the importance of studying microbial genomes to unravel fundamental mechanisms driving bacterial adaptation and survival in specific ecological niches like the GI tract.

### 3.6. Carbohydrate-Active Enzymes (CAZymes)

Carbohydrate-active enzymes (CAZymes) are a diverse group of enzymes encoded by the genome of the gut microbiota, and they play a crucial role in breaking down complex carbohydrates into simpler components [27]. These enzymes are essential for our nutrition as they facilitate the digestion and absorption of carbohydrates.

The CAZymes present in YLR001 encompass 49 genes distributed among four gene families (Table S4): Glycoside Hydrolases (GHs), Glycosyl Transferases (GTs), Carbohydrate Esterases (CEs), and Auxiliary Activities (AAs). Among these, there are 14 genes encoding Glycoside Hydrolases, which catalyze the hydrolysis of glycosidic bonds in carbohydrates. This process is vital for releasing glucose molecules from various sources of carbohydrates. Additionally, YLR001 contains 23 genes encoding Glycosyl Transferases. These enzymes participate in the biosynthesis of disaccharides, oligosaccharides, and polysaccharides by forming new glycosidic bonds between sugar molecules. The diversity of these glycosyltransferase genes highlights their importance in synthesizing complex carbohydrates found in exopolysaccharides or cell walls. Furthermore, YLR001 harbors 10 genes encoding Carbohydrate Esterase. These enzymes contribute to the breakdown of ester linkages within carbohydrate structures. By cleaving these ester bonds, Carbohydrate Esterase aids in further disintegrating complex carbohydrates into more manageable forms that our bodies can readily utilize. Two genes related to Auxiliary Activities have been identified within the YLR001 CAZyme repertoire. Although less understood than other CAZyme families, Auxiliary Activities encompass various enzymatic functions involved in carbohydrate metabolism beyond hydrolysis or synthesis reactions.

In summary, diverse CAZymes within the YLR001 genome underscore their indispensable role in carbohydrate degradation and utilization. Through their concerted actions on different types of chemical bonds present within carbohydrates, these enzymes enable us to derive energy and nutrients from a wide range of plant-based foods we consume daily.

### 3.7. Adaptation to Stress

Lactic acid bacteria (LAB) capable of surviving in the gastric environment typically possess stress response genes that confer adaptability to the intestinal environment and promote beneficial health effects [28,29]. Therefore, environment tolerance is a desirable characteristic of LAB, as they must endure diverse conditions such as variations in temperature, pH, and salinity. The genome sequence of *L. reuteri* YLR001 reveals several stress-related genes involved in resistance against low pH, bile salts, and heat stress (Table S5).

*L. reuteri* possesses genes encoding various stress-related proteins, including multiple proteases involved in stress response, such as energy-dependent intracellular proteases *clpP* (K01358; gene0454 and gene1715). These proteases exhibit chymotrypsin-like activity and play a significant role in the degradation of misfolded proteins. Moreover, the genome of *L. reuteri* YLR001 revealed the presence of genes encoding cold shock proteins (*CspA*, gene0744, and gene1803), which play a crucial role in nucleic acid-binding and serve as transcriptional regulators. And this strain also exhibited various heat shock proteins synthesized by microorganisms to adapt to environmental temperatures, including HSP20 (gene0698), HSP33 (gene0310), htpX (gene0276), Co-Chaperonin *groES* (gene0415), Molecular Chaperonin *groEL* (gene0416), Molecular Chaperonin *dank* (gene0876), and Molecular Chaperonin *dnaJ* (gene0877). Furthermore, as previously mentioned regarding *L. reuteri* WHH1689, the YLR001 also harbors the *hrcA* (gene0874) gene, which encodes a heat shock protein involved in DNA binding. The presence of *hrcA-grpE-dnaK-dnaJ* signifies adaptive responses to changes in environmental temperature. These findings indicate that YLR001 exhibits a strong ability to adapt to diverse ecological conditions. 

As is well known, stress-related proteins not only reveal genetic adaptation but also regulate evolution resistance [30]. In addition to other common stress pathways, lactic acid-producing bacteria must efficiently deal with the acidification of their local environment. F0F1-ATP synthase was recognized as the primary regulator of intracellular pH [31]. Within the YLR001 genome, eight genes (*atpA-atpH*; gene0022-gene0029) encoding different subunits of the F0F1-ATP synthase subunit were identified, along with five other genes (genes 0194, 0234, 0532, 0533, and 1969) encoding sodium-proton (Na^+^/H^+^) antiporters that play a central role in pH regulation and Na^+^ homeostasis. In addition, the YLR001 genome also revealed the presence of five genes associated with alkaline stress response proteins, including alkaline shock protein (gene0965 and gene1359), alkaline phosphatase (gene2087 and gene2130), and alkaline phosphatase family protein (gene2185).

Furthermore, it has been demonstrated that cyclopropane-fatty-acyl-phospholipid synthase and bile salt hydrolase (BSH) promote bile salt tolerance [31]. In the genome of *L. reuteri* YLR001, a gene cluster *cfa* (gene0142) associated with cyclopropane-fatty-acyl-phospholipid synthase has been identified, enhancing the resistance capability of *L. reuteri* YLR001 against bile salts. Moreover, the presence of *ppaC* (gene0960), encoding for manganese-dependent inorganic pyrophosphatase in YLR001 strains, plays a crucial role in maintaining membrane integrity and promoting tolerance to bile salts. Additionally, similar to *L. reuteri* ATCC53608, ZLR003, and YSJL-12 strains, the genome of YLR001 also encodes choloylglycine hydrolase (gene0904), which is implicated in combating bile stress. These findings suggest that strain YLR001 holds promise as a potential probiotic candidate for cholesterol reduction purposes.

According to the sequencing data analysis, YLR001 was found to harbor a diverse array of potential genes encoding antioxidant resistance. These genetic elements play pivotal roles in safeguarding cells against oxidative stress and upholding cellular redox equilibrium. One such gene is thioredoxin (gene1433), which serves as a pivotal regulator of cellular redox status by facilitating the reduction of disulfide bonds in proteins. Another significant gene, *fnr*, encodes for thioredoxin reductases (K21567, gene1961). Thioredoxin reductases are enzymatic catalysts responsible for replenishing active thioredoxins by reducing their disulfide bonds. In addition, YLR001 also harbors the trxA (K03671, gene0608, gene2141, and gene1513) and trxB (K00384, gene0438) genes. The former encodes for thiol reductase thioredoxin, while the latter encodes for thioredoxin-disulfide reductase. Both enzymes play a crucial role in maintaining protein reduction within cellular environments. Moreover, K03885 (gene0577) has been identified in the YLR001 genome. This gene encodes for NADH dehydrogenase and plays a vital role in energy production through electron transport chain reactions. Additionally, YLR001 exhibits an array of antioxidant defense mechanisms facilitated by enzymatic systems such as NADH oxidases (gene0077), NADH-flavin reductase (gene0192), NADH peroxidase Npx (gene1913, gene1914), and peptide-methionine(R)-S-oxide reductase (gene0219). These findings suggest that YLR001 possesses robust mechanisms to effectively counteract oxidative stress and maintain cellular homeostasis even under challenging environmental conditions.

The adhesion of microorganisms to epithelial cells is a complex process, as it largely depends on the chemical composition and physical properties of the cell surface of probiotic strains [32]. Probiotics can specifically bind to receptors on intestinal mucosal epithelial cells through hydrophobicity and potential surface exposure (PSE) proteins. Among these proteins, the PSE protein is crucial for adhering to the intestinal surface [33]. *L. reuteri* YLR001 encodes several well-known adhesion factors, including cell surface hydrolase (gene2153); ErfK family cell surface protein (gene2156); *lspA,* which encodes for lipoprotein signal peptidase (K03101, gene1049); *tuf,* which encodes for elongation factor Tu (K02358, gene0795); and TPI, which encodes for triosephosphate isomerase (K01803, gene0795). In line with previous research, *this* was associated with glycolysis, which the organism can utilize for acclimatization and enhancement of adhesion capability [18]. The findings indicate that YLR001 exhibits a higher abundance of genes encoding surface proteins and demonstrates superior performance in terms of adhesion activity.

The expression of genes encoding for antibacterial peptides and bacteriocin in LAB strains can provide them with the ability to combat bacterial infection [34]. In particular, strain YLR001 has been identified to harbor *agrA* (K07707) and *agrC* (K07706) genes responsible for bacteriocin synthesis. These genes play a pivotal role in inhibiting the growth of Gram-positive and Gram-negative bacteria, rendering YLR001 an efficacious defense mechanism against diverse types of detrimental bacteria.

Furthermore, the presence of antistress genes in *L. reuteri* YLR001 suggests that this strain may confer additional advantageous effects on the host. These anti-stress genes enable the LAB strain to withstand better environmental stressors, such as fluctuations in pH or temperature. YLR001 can persist longer within the host’s gastrointestinal tract and positively influence gut health by ensuring its viability under challenging conditions.

These findings underscore the therapeutic potential of LAB strains such as *L. reuteri* YLR001 in promoting a harmonious microbiota balance and safeguarding against bacterial infections. Further investigation is warranted to comprehensively elucidate their mechanisms of action and explore their prospective utilization as probiotics or antimicrobial agents in clinical settings.

### 3.8. Mobile Genetic Element Analysis

The *L. reuteri* YLR001 genome analysis was conducted for phage detection. PHAST, for PHAge Search Tool, was the tool applied in this study to detect prophages [35]. It revealed the presence of five intact prophage elements, which are genetic sequences that can integrate into a bacterial genome and replicate along with it. These prophages play an essential role in the evolution and adaptation of bacteria. The details of prophage characteristics are shown in Supplementary Materials (Table S6), which includes various attributes of each prophage element identified in the YLR001 genome.

Among these four prophages, two were similar to *Staphylococcus aureus* phages. The first one had a size of 27.4 kb and was located in Region 1, while the second one had a size of 15.4 kb in Region 2 (NC_009763). Both of these *Staphylococcus aureus* phages exhibited different GC contents, with one having a content of 37.64% and the other having a content of 36.5%. Another identified prophage belonged to the *Lactobacillus* phage path, which had a size of 36.7 kb and was located in Region 3 (NC-000896). This particular phage displayed a GC content of 37.38%. In addition, another prophage element resembled *Bacillus* phage Fah, with a size of 27.8 kb, also located in Region 3 (NC-007814). Interestingly, this Bacillus phage Fah-like element exhibited a higher GC content than the others, at approximately 43.18%. These findings highlight the diversity within the YLR001 genome regarding its integrated viral elements or prophages from different bacterial species, such as *Staphylococcus aureus* and *Lactobacillus* species, and those resembling Bacillus phages like Fah. Further studies on these intact prophage elements will contribute to our understanding of their potential impact on host bacterial physiology and pathogenicity mechanisms.

Integrases are enzymes that play a crucial role in the integration and excision of prophages within bacterial genomes [36]. These integrases serve as valuable markers for identifying prophage regions, which are genetic elements derived from bacteriophages that have been integrated into the host genome. This study identified three integrations (genes 0509, 0514, 0830, and 1321) of interest in the prophage regions 1, 2, and 3. Prophage region 1 spanned from position 523,254 to position 550,669 base pairs (bp) on the bacterial genome. It contained 31 CDs, with an intact prophage element extending from gene0491 to gene0520, where the integrase is located.

Similarly, prophage region two extended from position 830,554 to position 846,010 bp on the genome. This region carried 19 CDs and harbored a complete prophage element from gene0812 to gene0830, where another integrase was identified. Furthermore, our analysis revealed that there is also a third prophage region present in this bacterial genome. Prophage region 3 extended from position 1,280,563 to position 1,317,311bp and contained 47 CDs with a complete prophage element from gene 1275 to gene 1321, where a phage integrase was located. These findings suggest that multiple phages have integrated into different regions of this bacterial genome over time.

Interestingly, gene0520 encoding for transposase was also identified in the YLR001 genome. This finding is consistent with previous reports on *L. reuteri* YSJL-12, suggesting that this gene plays an important role in the adaptation of the genome to its environment [37]. Transposases are enzymes responsible for the movement of genetic material within a genome, and their presence indicates that rearrangements and transpositions have occurred in the genome over evolutionary time. These findings highlight the complexity and dynamism of bacterial genomes and their ability to remodel and restructure in response to environmental pressures.

Previous studies have provided evidence that the CRISPR-Cas system, which involves the collaboration of CRISPR and Cas proteins, functions as a unique immune system in organisms by defending against exogenous DNA infections [38]. The genome of *L. reuteri* YLR001 has been found to contain a single CRISPR locus located within its chromosome. Three distinct spacers and four direct repeat (DR) sequences are present within this locus. These spacers and DR sequences play crucial roles in the adaptive immunity conferred by the CRISPR-Cas system.

The presence of multiple spacers suggests that *L. reuteri* YLR001 has encountered various types of foreign DNA threats in its environment over time. Each spacer represents a memory of past encounters with specific exogenous genetic material, enabling *L. reuteri* YLR001 to mount an efficient defense response upon subsequent exposure to similar invaders. This discovery highlights the significance of the CRISPR-Cas system as an essential component of microbial immune systems. It shows how bacteria like *L. reuteri* YLR001 have evolved sophisticated mechanisms to protect themselves from potential genomic invasions.

Further research into these spacer sequences and their corresponding targets will provide valuable insights into understanding host–pathogen interactions. It may even pave the way for developing novel strategies for combating infectious diseases or engineering precise gene editing tools based on this fascinating natural defense mechanism.

## 4. Conclusions

*L. reuteri* YLR001, a strain with potential probiotic properties, has been found to possess antioxidative capacity and the ability to enhance intestinal epithelial barrier function and defense against pathogens. The current genomic analysis provides valuable information for exploring the functional implications of *L. reuteri* YLR001. This genome information can serve as a foundation for future research to understand how this strain interacts with its host and benefits human health. In conclusion, the comparative genomic analysis of *L. reuteri YLR001* sheds light on its unique gene features and genetic adaptation. Further investigations are necessary to confirm these findings and delve deeper into the mechanisms underlying its probiotic properties.

## Figures and Tables

**Table 1:** General features of the *L. reuteri* YLR001 genome.

Feature	Values	Plasmids		
		Plas1	Plas2	Plas3
Genome size (bp)	2,242,943	132,625	38,926	24,864
GC content (%)	38.84%	36.39%	39.26%	33.51%
Protein-coding genes (CDS)	2183	151	37	24
tRNAs	69	8	0	0
rRNA operons	18	0	0	0
sRNA	1	0	0	0

## Data Availability

All data generated or analyzed during this study are included in this published article.

## Supplementary Materials

The following supporting information can be downloaded at: https://www.mdpi.com/article/10.3390/microorganisms12081636/s1, Table S1: The red dotted line in the figure highlights the bacterial strain under observation; Table S2: Unique genes of YLR001 genome; Table S3: Transporters of YLR001 genome and plasmids; Table S4: Genes associated with four CAZymes gene families; Table S5: The antistress proteins of Lactobacillus reuteri YLR001 genome; Table S6: Complete prophage regions of YLR001 genome.
